# Comparative Evaluation of Surface Hardness, Roughness, Tensile Strength, and Antifungal Property of Soft Liners Incorporated With Silver Vanadate and Titanium Dioxide Nanoparticles: An In Vitro Study

**DOI:** 10.7759/cureus.95488

**Published:** 2025-10-27

**Authors:** Noveena Dhanalakshmi, Mathew Chalakuzhiyil Abraham, Vidhyasankari N, Rajkumar K, Mayavaraj R, Boopesh Rajendran

**Affiliations:** 1 Department of Prosthodontics, KSR Institute of Dental Science and Research, Tiruchengode, IND

**Keywords:** antifungal efficacy, roughness, silver vanadate, surface hardness, titanium dioxide

## Abstract

Aim

This in vitro study aimed to evaluate and compare the antifungal efficacy, surface hardness, roughness, and tensile bond strength of heat-cure acrylic resin with soft liners incorporated with silver vanadate and titanium dioxide nanoparticles.

Methods and materials

In the present study, 120 was the total sample size; a total of six groups (A1, A2, A3, B1, B2, and B3) were prepared, with each group consisting of 20 specimens. The specimens from each group were further subdivided into four categories to evaluate different parameters, namely, antifungal efficacy, surface hardness, surface roughness, and tensile bond strength. Thus, for each parameter, five specimens were allotted from every group (n = 5). Two soft liners - Dentsply Viscogel (Dentsply Sirona, York, PA) (Group A) and Maarc Dental Soft Liner (Maarc Dental, Thane, India) (Group B) - were used. Each group was subdivided into a control group and an experimental group. Specimens were fabricated using stainless steel molds (10 mm diameter × 2 mm thickness for testing antifungal efficacy, surface hardness, and roughness; 40 mm × 10 mm × 10 mm for testing tensile bond strength of heat-cure acrylic resin with soft liner). Antifungal activity against *Candida albicans* was assessed using Sabouraud’s dextrose agar. Surface hardness and roughness were analyzed using a Shore A Durometer profilometer. Tensile bond strength of heat-cured acrylic resin with soft liner was measured using a universal testing machine.

Results and conclusion

Incorporation of silver vanadate significantly enhanced the antifungal activity against *Candida albicans*, as indicated by a larger zone of inhibition (p < 0.05). Surface hardness significantly increased in the Maarc Dental Soft Liner with titanium dioxide (p < 0.05), whereas silver vanadate caused a significant reduction (p < 0.05). In Dentsply Sirona Viscogel, silver vanadate significantly improved hardness (p < 0.05). Both silver vanadate and titanium dioxide nanoparticles significantly reduced surface roughness in all tested soft liners (p < 0.05). Tensile bond strength was significantly highest in the Maarc Dental Soft Liner with silver vanadate (p < 0.05), while no significant change was observed in Dentsply Sirona Viscogel (p > 0.05).

## Introduction

Removable dentures are worn by the elderly for longer periods than needed, with minimal rest given to the denture-bearing tissues. The longer wearing period leads to irritation of the soft tissues, depriving them of blood supply and resorption of the supporting bony foundation. Consequently, the dentures tend to loosen, necessitating the use of materials such as tissue conditioners and adhesives to enhance retention [1].

Soft lining materials appear to be used between 1% and 5% of all complete mandibular dentures. Their use is indicated in various circumstances, but most commonly where the oral mucosa covering the denture-bearing area is locally or generally of inadequate thickness, or where the oral mucosa exhibits a reduced tolerance to the loads applied to it by the denture [2].

The tissue conditioners or short-term soft liners are commonly used as temporary liners for the treatment and conditioning of ill-fitting dentures, for provisional or diagnostic purposes, temporary relining of immediate dentures or immediate surgical splints, relining cleft palate speech aids, tissue conditioning during implant healing, and for functional impressions [3].

One of the problems encountered while using soft denture liners is the growth of microorganisms. Microorganisms initially adhere to the surface of the lining and then penetrate into the material. Although microorganisms have the potential to penetrate soft denture liners, this occurs infrequently in clinical situations, as they predominantly remain adhered to the surface. The use of soft lining materials can intensify the progress of fungal and bacterial growth, which is supported by environmental conditions under the denture and the structure of the materials [4].

Silver (Ag) has been well known for its antimicrobial characteristics and has a long history of application in medicine. It has a well-tolerated tissue response and a low toxicity profile. It is more toxic than many other metals against a broad spectrum of sessile bacteria and fungi that colonize plastic surfaces [5]. Titanium dioxide nanoparticles are thought to exert their antimicrobial effects primarily through the generation of reactive oxygen species (ROS), which can damage microbial cell walls, membranes, and DNA, although the exact mechanism remains under investigation.

Research has shown that incorporating silver and titanium nanoparticles into denture lining materials can significantly enhance their antifungal and antibacterial properties. Modification of soft lining materials with silver and titanium nanoparticles is beneficial due to their known fungicidal and bactericidal effects [6]. While silver and titanium dioxide (TiO₂) have both been explored independently for such purposes, there is a lack of comparative studies evaluating their effects, particularly silver vanadate (AgVO₃) versus titanium dioxide in soft denture liners.

This in vitro study aims to evaluate and compare the antifungal efficacy, surface hardness, surface roughness, and tensile bond strength of heat-cure acrylic resin lined with two commercially available soft liners (Maarc Dental Soft Liner and Dentsply Sirona Viscogel) modified with either silver vanadate (AgVO₃) or titanium dioxide (TiO₂) nanoparticles.

## Materials and methods

Study design

This study followed an in vitro design to evaluate and compare the antifungal efficacy, surface hardness, roughness, and tensile bond strength of heat-cure acrylic resin with soft liners (Maarc Dental Soft Liner, Dentsply Sirona Viscogel) incorporated with silver vanadate (AgVO₃) and titanium dioxide (TiO₂) nanoparticles.

Study setting

The study was conducted at the KSR Institute of Dental Science and Research, as well as at Genolite Research and Development Laboratory, Coimbatore, Saveetha Dental College and Hospital, National Institute of Technology, Tiruchirappalli, and LMP Research and Development Laboratory, Pallipalayam. The laboratory facilities of the above institutions were utilized for preparing the samples of soft liner and heat-cure acrylic resin specimens with soft liner, testing, and evaluating the prepared samples for antifungal efficacy, roughness, hardness, and tensile bond strength. This study was carried out over a duration of four months to evaluate and compare the antimicrobial efficacy and mechanical properties of silver and titanium nanoparticle-modified soft denture liners.

Product names

The materials used in this study included MAARC Dental Soft Liner (Shiva Products, Gurugram, India) and Visco Gel Temporary Soft Denture Liner (Dentsply Caulk, Charlotte, NC) as the soft liners. The heat-cure denture base material was obtained from Dental Products of India (Mumbai, India). In addition, silver vanadate nanoparticles and titanium dioxide nanoparticles were procured from Nano Research Lab (Jamshedpur, India), a nanotechnology product. 

Sample size estimation 

The sample size was estimated using a t-test for the difference between two independent means (two groups). An a priori analysis was conducted to compute the required sample size, with the following parameters: one-tailed test, effect size (d) = 0.46, α error probability = 0.05, power (1-β error probability) = 0.8, and allocation ratio (N₂/N₁) = 1. The output yielded a noncentrality parameter (δ) of 2.5195, a critical t value of 1.6579, and an actual power of 0.8052, with degrees of freedom (df) = 118. Accordingly, each group comprised 60 samples, resulting in a total sample size of 120.

The sample size estimation was done based on the study by Kreve et al. [8], with an effect size of 0.46, and a power of 0.8.

Study flow diagram

**Figure 1:**
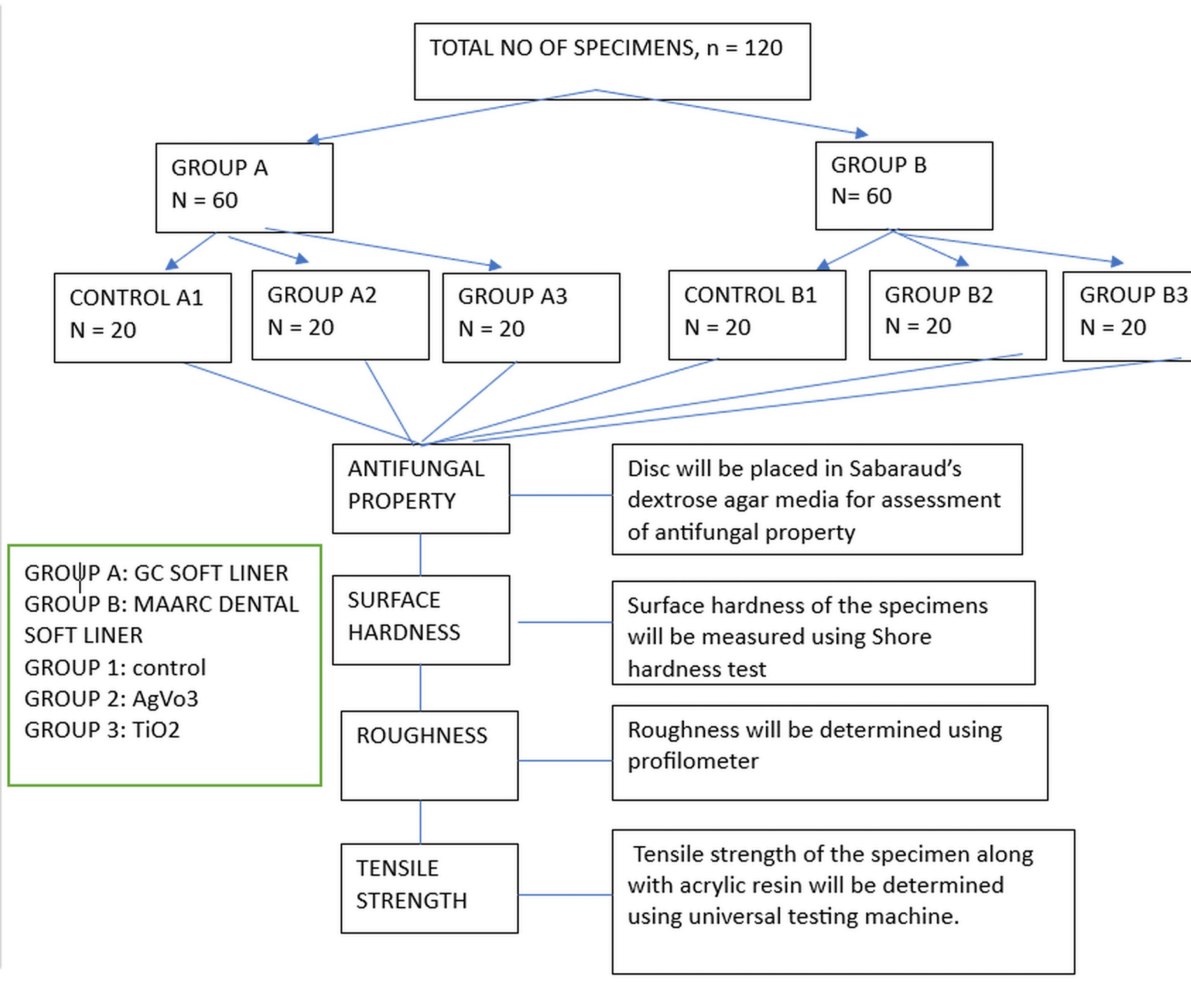
Study diagram

The study flow diagram is shown in Figure 1.

A total of 120 specimens were divided equally into two main groups, i.e., Group A and Group B, based on the type of soft liner material used. Group A consisted of 60 specimens fabricated using Maarc Dental Soft Liner. This group was further subdivided into three subgroups. Group A1, comprising 20 specimens, served as the control group and included soft liner specimens fabricated without the incorporation of nanoparticles. Group A2 included 20 specimens of Maarc Dental Soft Liner incorporated with 10% silver vanadate nanoparticles, while Group A3 consisted of 20 specimens incorporated with 10% titanium dioxide nanoparticles. Similarly, Group B consisted of 60 specimens fabricated using Viscogel Soft Liner and was also subdivided into three subgroups. Group B1, the control group, included 20 specimens of Viscogel Soft Liner without any nanoparticles. Group B2 included 20 Viscogel Soft Liner specimens incorporated with 10% silver vanadate nanoparticles, and Group B3 comprised 20 specimens incorporated with 10% titanium dioxide nanoparticles.

The proportion of nanoparticles incorporated into the soft liner was determined following a comprehensive literature review, which revealed that 10% concentrations of silver vanadate and titanium dioxide nanoparticles offer significant antimicrobial effects against *Candida albicans* [7]. A pilot study was conducted to determine the appropriate amount of nanoparticles to incorporate into each soft liner specimen. A single disc-shaped specimen required 0.4 g of soft liner for standardization. Accordingly, 0.04 g of nanoparticles (10% w/w) was incorporated into each specimen. Five specimens (n = 5) were used in the pilot study to ensure consistency and verify that this amount could be uniformly mixed into the soft liner before proceeding to the main experiment.

Based on this, 0.04 g (10% of 0.4 g) of nanoparticles were incorporated into each specimen. The mixing process was carried out using an ultrasonic processor (sonicator). Ultrasonication was employed to enhance nanoparticle distribution; however, minor agglomeration cannot be entirely ruled out. Despite this, the pilot study and specimen preparation were done to minimize variability, and all specimens were carefully mixed to achieve as uniform a distribution as possible. While higher nanoparticle concentrations could influence marginal integrity, 10% was chosen in this study to achieve significant antimicrobial effects without compromising the material’s functional properties.

For specimen fabrication, a stainless-steel mold measuring 10 mm in diameter and 2 mm in thickness was used. The soft liner material was manipulated according to the manufacturer's instructions, packed into the mold space using a plastic filling instrument, and compressed between two glass slabs to remove excess material. After polymerization, the specimens were carefully retrieved and examined for surface defects such as voids or irregularities; any flawed specimens were excluded from further testing.

To evaluate the tensile bond strength of heat-cured acrylic resin with the soft liner, additional specimens were fabricated using a stainless-steel die measuring 40 mm × 10 mm × 10 mm. These dies were invested in the lower half of a dental flask using type III dental stone, positioned to ensure even spacing and proper alignment. After the initial pour set, a separating medium (Cold Mold Seal) was applied, and the upper half of the flask was filled with another pour of dental stone. Following a 30-minute setting period, the dies were removed, and the mold space was prepared for acrylic resin processing. Heat-cure acrylic resin (DPI) was mixed as per the manufacturer’s guidelines, packed at the dough stage, and processed using a short curing cycle (74°C for two hours followed by 100°C for one hour). The flasks were allowed to cool to room temperature before specimens were retrieved, finished with 200-, 400-, and 600-grit sandpapers, and polished using a pumice slurry and buff wheel. A 3 mm wax spacer was placed between two heat-cured acrylic resin specimens to allow space for the soft liner. This assembly was invested in dental plaster in a flask, and after setting, dewaxing was performed at 98°C to eliminate the wax layer. The soft liner material was then packed into the space between the two acrylic blocks and polymerized. Upon setting, specimens with the soft liner interposed between acrylic sections were retrieved for testing tensile bond strength.

To assess the antifungal efficacy of the soft liners incorporated with silver vanadate and titanium dioxide nanoparticles, Sabouraud's dextrose agar plates were prepared and incubated at 37°C for 24 hours. A lawn culture of *Candida albicans* was developed on the agar, and the prepared soft liner specimens were placed on the surface to observe the zone of inhibition formed. The size of the inhibition zone (in mm) served as an indicator of antifungal activity. Surface hardness of the disc specimens was evaluated using a Shore A Durometer mounted on a hardness tester stand. The sample was positioned at a distance of 20 mm from the indenter, and readings were taken five seconds after penetration. Due to the inherent cushioning property of soft liners, a range of Shore A hardness values was observed. Surface roughness was assessed using a profilometer by recording three measurements per specimen, i.e., one at the center and two 1 mm adjacent to it, and the average value was calculated. Tensile bond strength testing was conducted using a universal testing machine (Kalpak Instruments and Controls, Puna, India) at a crosshead speed of 5 mm/minute. The peak load at failure (F) and the cross-sectional area (A) were recorded to calculate tensile stress (σ) using the formula σ = F/A. The operator was calibrated prior to the study through standardized training to ensure procedural consistency. Intra-operator reliability was verified by repeating measurements on randomly selected specimens.

Statistical analysis

Data regarding antifungal activity (zone of inhibition in mm), surface hardness (Vickers hardness number (VHN)), roughness (RA), and Tensile strength (N/mm2) in two experimental groups Maarc Dental Soft Liner and Dentsply Sirona Viscogel, and in three subgroups, control, AgVO₃, and TiO₂, were entered into Microsoft Excel (Microsoft® Corp., Redmond, WA) and analyzed using IBM SPSS Statistics for Windows Version 20 (IBM Corp., Armonk, NY). Data were investigated for normality using the Kolmogorov-Smirnov test. All parameters were checked for normal distribution. Descriptive statistics were expressed as mean, standard deviation (SD), and 95% confidence interval (CI). One-way analysis of variance (ANOVA) was performed to compare groups, and a p-value < 0.05 was considered statistically significant.

## Results

**Figure 2:**
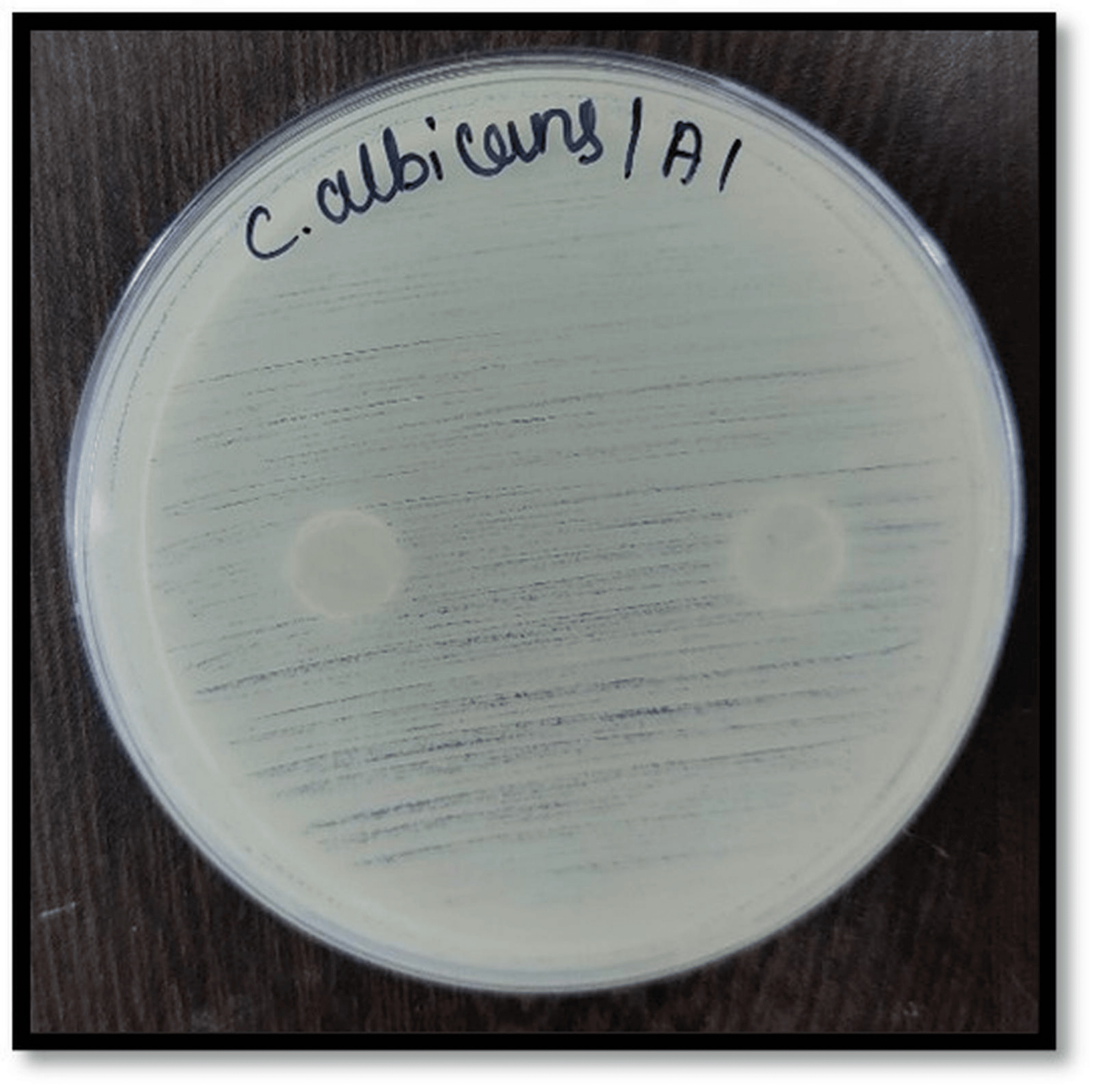
Zone of inhibition against Candida albicans for specimens of group A1 Group A1: Maarc Dental Soft Liner without incorporation of nanoparticles.

**Figure 3:**
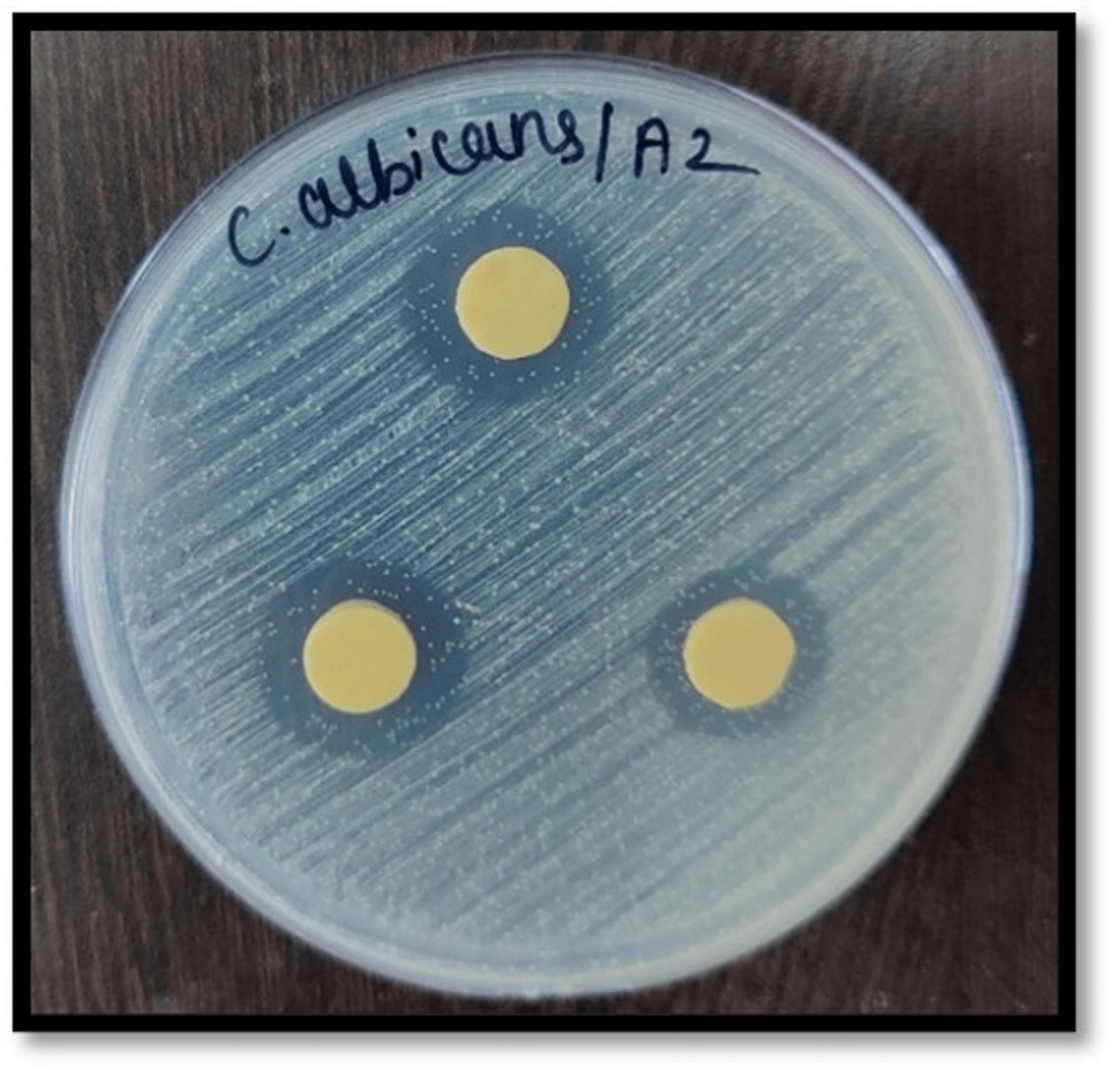
Zone of inhibition against Candida albicans for specimens of group A2 Group A2: Maarc Dental Soft Liner incorporated with silver vanadate nanoparticles.

**Figure 4:**
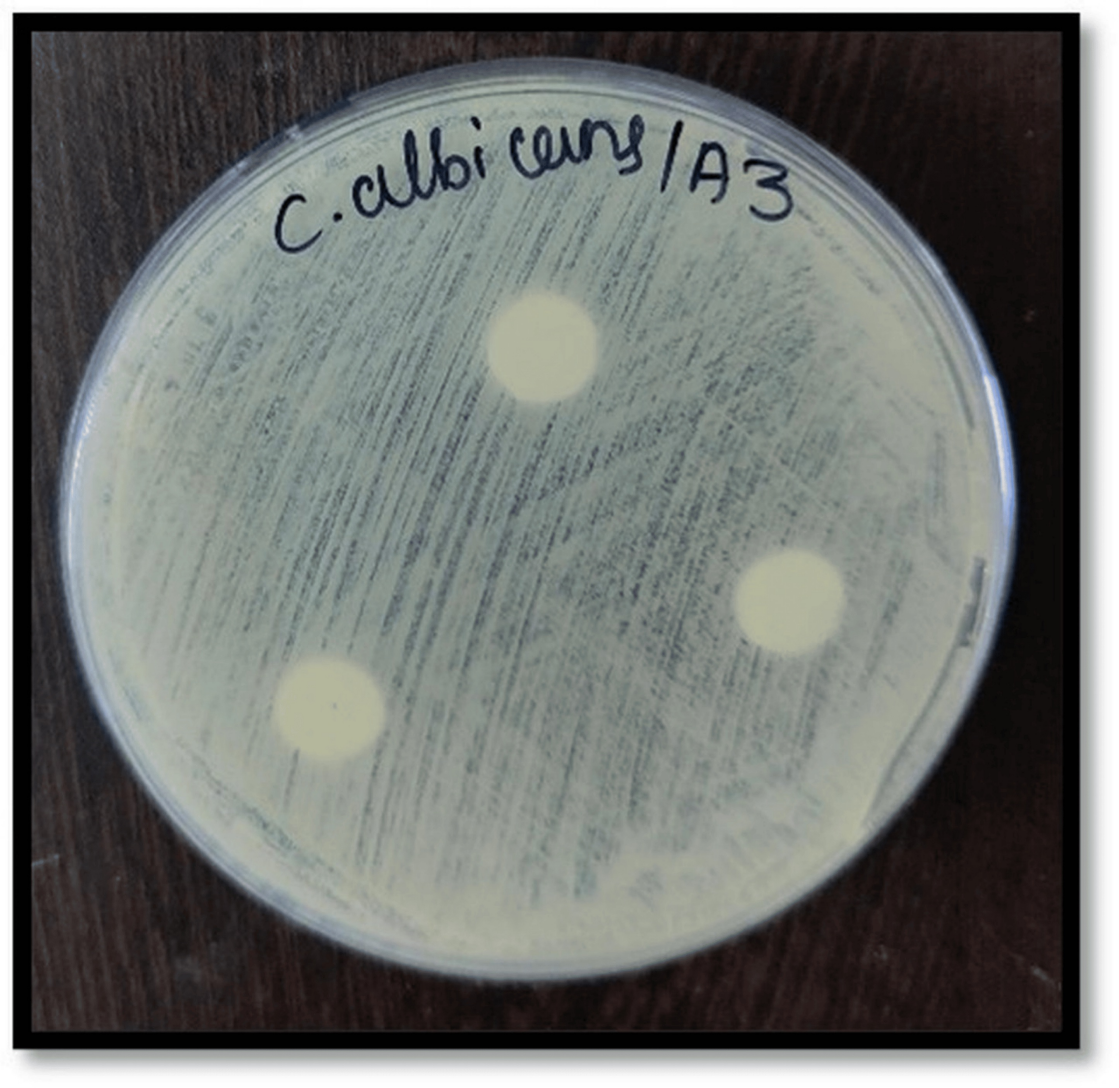
Zone of inhibition against Candida albicans for specimens of group A3 Group A3: Maarc Dental Soft Liner incorporated with titanium dioxide nanoparticles.

**Figure 5:**
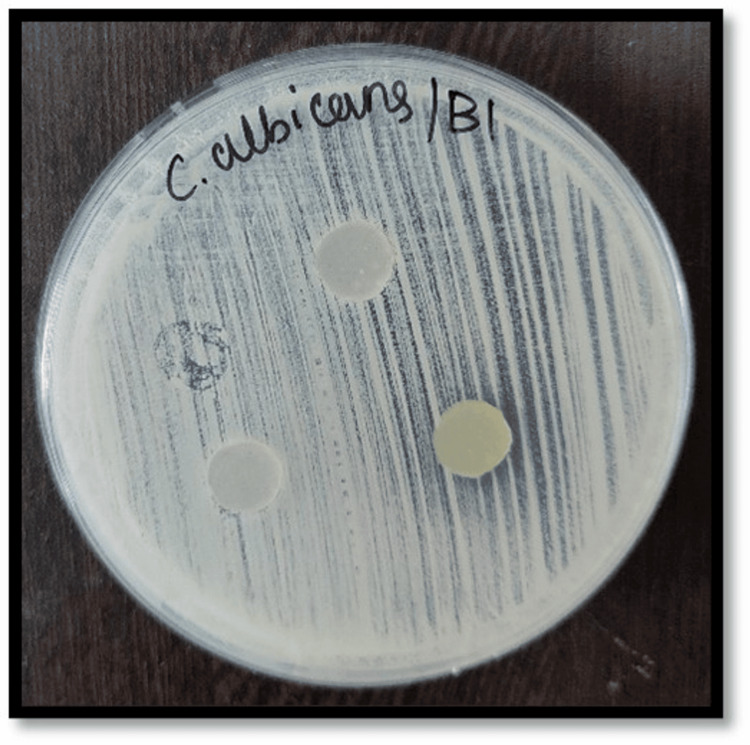
Zone of inhibition against Candida albicans for specimens of group B1 Group B1: Dentsply Sirona Viscogel without incorporation of nanoparticles.

**Figure 6:**
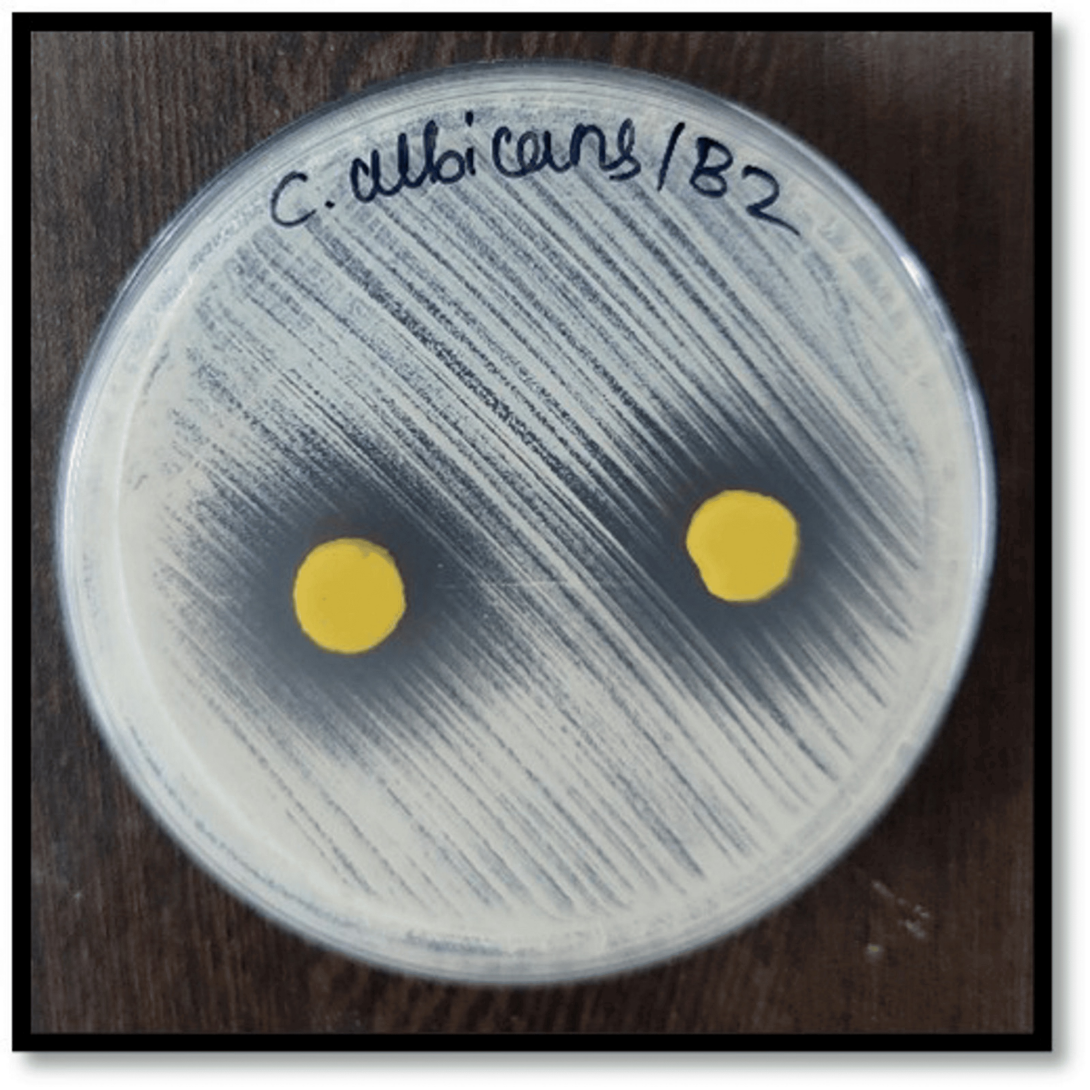
Zone of inhibition against Candida albicans for specimens of group B2 Group B2: Dentsply Sirona Viscogel incorporated with silver vanadate nanoparticles.

**Figure 7:**
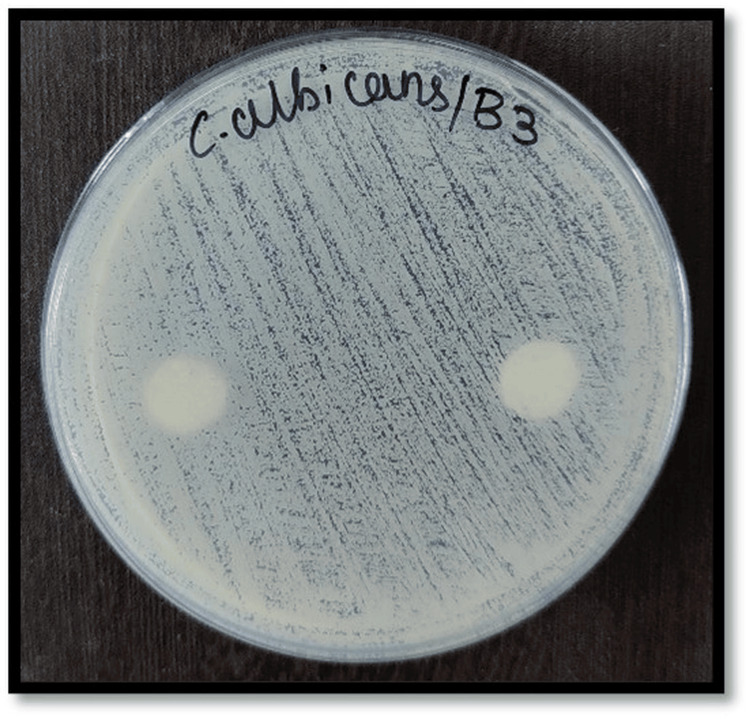
Zone of inhibition against Candida albicans for specimens of group B3 Group B3: Dentsply Sirona Viscogel incorporated with titanium dioxide nanoparticles.

Figures 2-7 depict the zones of inhibition formed against *Candida albicans* for each specimen group (A1, A2, A3, B1, B2, and B3). The zone of inhibition test revealed that only the AgVO₃ modified groups exhibited antimicrobial activity in both soft liners. The Maarc Dental Soft Liner with AgVO₃ showed a mean inhibition zone of 6.20 ± 2.58 mm, while Dentsply Sirona Viscogel with AgVO₃ demonstrated a higher inhibition zone of 13.60 ± 3.57 mm. Control and TiO₂ groups showed no inhibition zones in either material (Table 1).

**Table 1:** Mean, standard deviation, and 95% confidence interval for zone of inhibition (mm) AgVO₃ - silver vanadate; TiO₂ - titanium dioxide

Zone of Inhibition (mm)		n	Mean ± SD	Standard Error	95% Confidence Interval for Mean	
					Lower Bound	Upper Bound
Maarc Dental Soft Liner	Control	5	0.00 ± 0.00	0	0	0
	AgVO₃	5	6.20 ± 2.58	1.15	2.98	9.41
	TiO₂	5	0.00 ± 0.00	0	0	0
	Total	15	2.06 ± 3.32	0.85	0.22	3.9
Dentsply Sirona Viscogel	Control	5	0.00 ± 0.00	0	0	0
	AgVO₃	5	13.60 ± 3.57	1.6	9.15	18.04
	TiO₂	5	0.00 ± 0.00	0	0	0
	Total	15	4.53 ± 6.90	1.78	0.7	8.35

In terms of surface hardness, TiO₂ increased the hardness in Maarc (56.30 ± 3.53) compared to the control (55.05 ± 1.56), whereas AgVO₃ slightly reduced it (50.40 ± 3.01). For Viscogel, AgVO₃ significantly enhanced hardness (48.30 ± 2.06) compared to both control (41.70 ± 2.75) and TiO₂ (43.00 ± 1.58) (Table 2).

**Table 2:** Mean, standard deviation, and 95% confidence interval for surface hardness (VHN) VHN - Vickers hardness number

Surface Hardness (VHN)		n	Mean ± SD	Standard Error	95% Confidence Interval for Mean	
					Lower Bound	Upper Bound
Maarc Dental Soft Liner	Control	5	55.05 ± 1.56	0.7	53.1	56.99
	AgVO₃	5	50.40 ± 3.01	1.34	46.65	54.14
	TiO₂	5	56.30 ± 3.53	1.57	51.91	60.68
	Total	15	53.91 ± 3.71	0.95	51.86	55.97
Dentsply Sirona Viscogel	Control	5	41.70 ± 2.75	1.23	38.27	45.12
	AgVO₃	5	48.30 ± 2.06	0.92	45.73	50.86
	TiO₂	5	43.00 ± 1.58	0.7	41.03	44.96
	Total	15	44.33 ± 3.58	0.92	42.34	46.31

Surface roughness was lowest in the AgVO₃ groups for both materials (Maarc: 0.33 ± 0.30; Viscogel: 1.59 ± 0.22), while control groups had the highest roughness. TiO₂ moderately reduced roughness compared to the control (Table 3).

**Table 3:** Mean, standard deviation, and 95% confidence interval for roughness (Ra) Ra - roughness average

Roughness (Ra)		n	Mean ± SD	Standard Error	95% Confidence Interval for Mean	
					Lower Bound	Upper Bound
Maarc Dental Soft Liner	Control	5	5.07 ± 0.76	0.34	4.11	6.03
	AgVO₃	5	0.33 ± 0.30	0.13	-0.03	0.71
	TiO₂	5	1.49 ± 0.53	0.23	0.83	2.15
	Total	15	2.30 ± 2.15	0.55	1.11	3.49
Dentsply Sirona Viscogel	Control	5	5.31 ± 1.33	0.59	3.65	6.97
	AgVO₃	5	1.59 ± 0.22	0.1	1.31	1.87
	TiO₂	5	1.59 ± 0.37	0.16	1.12	2.06
	Total	15	2.83 ± 1.96	0.5	1.74	3.92

Regarding tensile bond strength, the AgVO₃ group in Maarc showed the highest value (0.178 ± 0.014 N/mm²), outperforming the control (0.089 ± 0.007) and TiO₂ (0.067 ± 0.006). In contrast, for Viscogel, the control group exhibited the highest bond strength (0.186 ± 0.041), while AgVO₃ and TiO₂ groups had lower values (0.118 ± 0.006 and 0.137 ± 0.034, respectively) (Table 4).

**Table 4:** Mean, standard deviation, and 95% confidence interval for tensile bond strength of heat-cured acrylic resin with soft liner (N/mm2) N/mm^2 ^- Newton per square millimeter

Tensile Bond Strength (N/mm^2^)		n	Mean ± SD	Standard Error	95% Confidence Interval for Mean	
					Lower Bound	Upper Bound
Maarc Dental Soft Liner	Control	5	0.089 ± 0.007	0.003	0.076	0.098
	AgVO₃	5	0.178 ± 0.014	0.006	0.16	0.196
	TiO₂	5	0.067 ± 0.006	0.002	0.059	0.075
	Total	15	0.111 ± 0.050	0.013	0.083	0.139
Dentsply Sirona Viscogel	Control	5	0.186 ± 0.041	0.018	0.134	0.2383
	AgVO₃	5	0.118 ± 0.006	0.002	0.11	0.125
	TiO₂	5	0.137 ± 0.034	0.015	0.094	0.18
	Total	15	0.147 ± 0.041	0.01	0.124	0.17

In the present study, a total of six groups (A1, A2, A3, B1, B2, and B3) were prepared, with each group consisting of 20 specimens. The specimens from each group were further subdivided into four categories to evaluate different parameters, namely antifungal efficacy, surface hardness, surface roughness, and tensile bond strength. Thus, for each parameter, five specimens were allotted from every group (n = 5). The sample size estimation was done based on the study by Kreve et al. [8], with an effect size of 0.46, and a power of 0.8. Accordingly, for each test parameter, a total of 30 specimens (six groups × five specimens) were analyzed, making the overall sample size 120 specimens (six groups × 20 specimens). Tables 5-8 depict a one-way ANOVA test for all the parameters, and Tables 9-12 depict post hoc analysis.

**Table 5:** One-way ANOVA test for zone of inhibition

Zone of Inhibition (mm)		Sum of Squares	df	Mean Square	F	p-value
Maarc Dental Soft Liner	Between groups	128.133	2	64.067	28.687	0.000
	Within groups	26.8	12	2.233		
	Total	154.933	14			
Dentsply Sirona Viscogel	Between groups	616.533	2	308.267	72.25	0.000
	Within groups	51.2	12	4.267		
	Total	667.733	14			

**Table 6:** One-way ANOVA test for surface hardness

Surface Hardness (VHN)		Sum of Squares	df	Mean Square	F	p-value
Maarc Dental Soft Liner	Between groups	96.658	2	48.329	6.038	0.015*
	Within groups	96.05	12	8.004		
	Total	192.708	14			
Dentsply Sirona Viscogel	Between groups	122.233	2	61.117	12.76	0.001*
	Within groups	57.475	12	4.79		
	Total	179.708	14			

**Table 7:** One-way ANOVA test for roughness

Roughness (Ra)		Sum of Squares	df	Mean Square	F	p-value
Maarc Dental Soft Liner	Between groups	60.966	2	30.483	94.331	0.000*
	Within groups	3.878	12	0.323		
	Total	64.844	14			
Dentsply Sirona Viscogel	Between groups	46.111	2	23.055	34.937	0.000*
	Within groups	7.919	12	0.66		
	Total	54.029	14			

**Table 8:** One-way ANOVA test for tensile bond strength

Tensile Bond Strength (N/mm^2^)		Sum of Squares	df	Mean Square	F	p-value
Maarc Dental Soft Liner	Between groups	0.035	2	0.017	169.938	0.000*
	Within groups	0.001	12	0		
	Total	0.036	14			
Dentsply Sirona Viscogel	Between groups	0.012	2	0.006	6.279	0.014*
	Within groups	0.012	12	0.001		
	Total	0.024	14			

**Table 9:** Multiple comparisons: Tukey HSD for zone of inhibition (mm)

Dependent Variable			Mean Difference (I-J)	Standard Error	p-value	95% Confidence Interval	
						Lower Bound	Upper Bound
Maarc Dental Soft Liner	Control	AgVO₃	-6.200^*^	0.945	0	-8.721	-3.678
		TiO₂	0	0.945	1	-2.521	2.521
	AgVO₃	Control	6.200^*^	0.945	0	3.678	8.721
		TiO₂	6.200^*^	0.945	0	3.678	8.721
	TiO₂	Control	0	0.945	1	-2.521	2.521
		AgVO₃	-6.200^*^	0.945	0	-8.721	-3.678
Dentsply Sirona Viscogel	Control	AgVO₃	-13.600^*^	1.306	0	-17.085	-10.114
		TiO₂	0	1.306	1	-3.485	3.485
	AgVO₃	Control	13.600^*^	1.306	0	10.114	17.085
		TiO₂	13.600^*^	1.306	0	10.114	17.085
	TiO₂	Control	0	1.306	1	-3.485	3.485
		AgVO₃	-13.600^*^	1.306	0	-17.085	-10.114

**Table 10:** Multiple comparisons: Tukey HSD for surface hardness (VHN) VHN: Vickers hardness number

Dependent Variable			Mean Difference (I-J)	Standard Error	p-value	95% Confidence Interval	
						Lower Bound	Upper Bound
Maarc Dental Soft Liner	Control	AgVO₃	4.65	1.789	0.056	-0.123	9.423
		TiO₂	-1.25	1.789	0.769	-6.023	3.523
	AgVO₃	Control	-4.65	1.789	0.056	-9.423	0.123
		TiO₂	-5.900^*^	1.789	0.016	-10.673	-1.126
	TiO₂	Control	1.25	1.789	0.769	-3.523	6.023
		AgVO₃	5.900^*^	1.789	0.016	1.126	10.673
Dentsply Sirona Viscogel	Control	AgVO₃	-6.600^*^	1.384	0.001	-10.292	-2.907
		TiO₂	-1.3	1.384	0.627	-4.992	2.392
	AgVO₃	Control	6.600^*^	1.384	0.001	2.907	10.292
		TiO₂	5.300^*^	1.384	0.006	1.607	8.992
	TiO₂	Control	1.3	1.384	0.627	-2.392	4.992
		AgVO₃	-5.300^*^	1.384	0.006	-8.992	-1.607

**Table 11:** Multiple comparisons: Tukey HSD for roughness (Ra) Ra: roughness average

Dependent Variable			Mean Difference (I-J)	Standard Error	p-value	95% Confidence Interval	
						Lower Bound	Upper Bound
Maarc Dental Soft Liner	Control	AgVO₃	4.735^*^	0.359	0	3.776	5.694
		TiO₂	3.581^*^	0.359	0	2.622	4.54
	AgVO₃	Control	-4.735^*^	0.359	0	-5.694	-3.776
		TiO₂	-1.153^*^	0.359	0.019	-2.113	-0.194
	TiO₂	Control	-3.581^*^	0.359	0	-4.54	-2.622
		AgVO₃	1.153^*^	0.359	0.019	0.194	2.113
Dentsply Sirona Viscogel	Control	AgVO₃	3.719^*^	0.513	0	2.349	5.09
		TiO₂	3.718^*^	0.513	0	2.348	5.089
	AgVO₃	Control	-3.719^*^	0.513	0	-5.09	-2.349
		TiO₂	-0.001	0.513	1	-1.371	1.369
	TiO₂	Control	-3.718^*^	0.513	0	-5.089	-2.348
		AgVO₃	0.001	0.513	1	-1.369	1.371

**Table 12:** Multiple comparisons: Tukey HSD for tensile bond strength (N/mm2)

Dependent Variable	Group A	Group A	Mean Difference	Standard Error	p-value	95% Confidence Interval	
						Lower Bound	Upper Bound
Maarc Dental Soft Liner	Control	AgVO₃	-0.08920^*^	0.00638	0	-0.1062	-0.0722
		TiO₂	0.02180^*^	0.00638	0.013	0.0048	0.0388
	AgVO₃	Control	0.08920^*^	0.00638	0	0.0722	0.1062
		TiO₂	0.11100^*^	0.00638	0	0.094	0.128
	TiO₂	Control	-0.02180^*^	0.00638	0.013	-0.0388	-0.0048
		AgVO₃	-0.11100^*^	0.00638	0	-0.128	-0.094
Dentsply Sirona Viscogel	Control	AgVO₃	0.06840^*^	0.0199	0.013	0.0153	0.1215
		TiO₂	0.049	0.0199	0.071	-0.0041	0.1021
	AgVO₃	Control	-0.06840^*^	0.0199	0.013	-0.1215	-0.0153
		TiO₂	-0.0194	0.0199	0.606	-0.0725	0.0337
	TiO₂	Control	-0.049	0.0199	0.071	-0.1021	0.0041
		AgVO₃	0.0194	0.0199	0.606	-0.0337	0.0725

## Discussion

Soft lining materials are primarily used for patients who cannot tolerate the pressure exerted by a prosthesis on the mucosa of the edentulous ridge [7]. Tissue conditioners and temporary soft lining materials tend to harden with extended use. As this occurs, the material's surface becomes rough and irregular, thereby increasing the risk of tissue trauma. Furthermore, in their hardened form, these materials are more susceptible to colonization by *Candida* species, increasing the likelihood of developing denture-induced stomatitis in patients [4]. It has been reported that denture disinfectants, such as chlorhexidine gluconate, sodium hypochlorite, and hydrogen peroxide, can cause adverse changes to the physical and chemical properties of soft liners [3]. Research has shown that incorporating silver and titanium nanoparticles into denture lining materials can significantly enhance their antifungal and antibacterial properties [4].

Kreve et al. [8] evaluated the antimicrobial properties of denture liners modified with silver vanadate using the Kirby-Bauer agar diffusion method. They found that lower concentrations (1% and 2.5%) were ineffective, while 5% and 10% concentrations showed antimicrobial activity against *Pseudomonas aeruginosa*, with the 10% concentration also demonstrating superior effectiveness against *Candida albicans* [8]. Shibata et al. in their study evaluated the antifungal effectiveness of acrylic resin modified with apatite-coated titanium dioxide, demonstrating a notable reduction in the viability of *Candida albicans* when the material contained 5% and 10% titanium dioxide by weight [9]. Hence, in our study, a 10% concentration of silver vanadate and titanium dioxide was incorporated to test the antifungal efficacy of the soft liner against *Candida albicans*. The zone of inhibition was measured against *Candida albicans* in Sabouraud's dextrose agar, where 10% silver vanadate was incorporated in Dentsply Sirona Viscogel and Maarc Dental Soft Liner . In our study, Dentsply Sirona Viscogel incorporated with 10% silver vanadate showed a higher zone of inhibition than Maarc Dental Soft Liner incorporated with 10% silver vanadate (Figures 1-6). This aligns with findings by Deng et al. [10], where in situ-synthesized silver nanoparticles in denture liners enhanced antifungal activity due to the uniform distribution of nanoparticles. Similarly, the superior performance of Dentsply Viscogel may have resulted from better nanoparticle dispersion and compatibility with the material, leading to more effective ROS generation, membrane disruption, and silver ion release [10]. In the present study, no specific light activation or photocatalytic conditions were applied; hence, the observed antimicrobial effect can be attributed to the direct release of silver ions and subsequent disruption of microbial cell membranes rather than ROS-mediated mechanisms. In contrast to our findings with 10% silver vanadate, where both Dentsply Sirona Viscogel and Maarc Dental Soft Liners exhibited clear zones of inhibition, indicating notable antimicrobial activity, 10% titanium dioxide-incorporated soft liners showed no zone of inhibition in our study. This aligns with the results reported by Neppala et al., where titanium nanoparticles demonstrated limited or no antifungal efficacy against *Candida albicans* and *Streptococcus mutans*, and only moderate activity against *Lactobacillus* [11]. These comparisons highlight that silver vanadate exhibits broader and more potent antimicrobial effects, particularly against fungal and bacterial pathogens relevant to oral health, reinforcing its potential as a more effective additive for denture soft liners than titanium dioxide. The reason for the absence of a zone of inhibition against *Candida albicans* may be due to the photocatalytic nature of titanium dioxide nanoparticles, which require activation by ultraviolet light to produce ROS, responsible for antifungal activity [11]. In our study, due to the absence of UV light during the in vitro testing, 10% titanium dioxide incorporated in both Maarc Dental Soft Liner and Dentsply Sirona Viscogel remained inactive, resulting in limited or no antifungal effect against *Candida albicans*. 

This discrepancy may be attributed to nanoparticle agglomeration and altered polymer-nanoparticle interactions at higher concentrations, which create weak zones within the matrix and compromise stress distribution, underscoring the necessity of optimizing nanoparticle content to achieve a balance between antimicrobial efficacy and mechanical integrity.

Soft liner with decreased hardness is preferable because it will have greater softness and thus increased ability to act as an absorbing cushion for occlusal forces. Soft denture liners should exhibit low hardness levels; however, during use, they are susceptible to changes related to the leaching of plasticizers [8,12]. Kreve et al. found that the hardness of soft lining materials increased with age due to the hydrophilic character of the material, which can lead to hardening through ethanol loss, water absorption, and loss of the plasticizer [8]. In our study, the Maarc Dental Soft Liner incorporated with 10% silver vanadate nanoparticles showed the lowest surface hardness compared to both the control and the group with 10% titanium dioxide. This contrasts with the study by Ahmed et al. [12], which reported a notable reduction in surface hardness in soft liners with titanium nanoparticles. Their scanning electron microscopy analysis revealed poor nanoparticle dispersion due to Van der Waals forces, leading to incomplete polymer conversion and increased residual monomer, which acts as a plasticizer and thereby reduces the hardness of the soft liner [12]. As residual monomer content was not measured in this study, its influence on mechanical properties remains uncertain, with the observed hardness variations likely arising from nanoparticle interactions, possible agglomeration, and polymer matrix alterations, warranting future quantification of residual monomer levels to elucidate its role in soft liner mechanics. In our study, the incorporation of 10% silver vanadate nanoparticles into Maarc Dental Soft Liner resulted in the lowest surface hardness. This has been compared with findings by Kreve et al. [8], who reported that adding 1% silver vanadate to soft liners yielded hardness values that remained within the acceptable range defined by ISO 10139-2:2009 for resilient denture liners [8]. Urban et al. [13] stated that the antimicrobial agents dispersed inside the soft liner in gel form may also act as fillers, increasing their resistance and thus their hardness. Hence, when a soft liner is incorporated with nanoparticles in a powder form, it decreases its hardness. In the present study, 10% silver vanadate and 10% titanium dioxide nanoparticles have been incorporated in the polymer of the soft liner in powdered form; hence, there was a decrease in hardness [13].

Soft liners undergo leaching of plasticizers and release of monomers over time, which increases the stiffness and roughness of the material. In patients with existing denture stomatitis, the roughness of the denture liner increases significantly due to the release of products such as protease, lipase, phospholipase, and acids from microorganisms [14]. In the study by Kreve et al., the incorporation of different concentrations of silver vanadate nanoparticles did not affect the roughness of Trusoft soft liner [8]. In our study, experimental groups of Maarc Dental Soft Liner and Dentsply Sirona Viscogel incorporated with 10% Silver Vanadate and 10% Titanium Dioxide demonstrated decreased roughness compared to the control groups of both soft liners. The reduction in roughness of the soft liners incorporated with nanoparticles is beneficial, as it may lead to reduced microbial adhesion, thereby decreasing the risk of denture stomatitis.

The study conducted by Naji et al. [15] reported a reduction in surface roughness in specimens reinforced with zirconium dioxide and titanium dioxide nanoparticles when compared to the control group. This reduction may be attributed to the fact that surface roughness measurements primarily assess the external surface. The nanoparticles, owing to their ultra-fine size, were uniformly dispersed within the polymer matrix, minimizing agglomeration and enhancing interfacial bonding between the soft liner and the polymer substrate [15]. An increase in surface roughness can lead to greater bacterial adhesion, which is undesirable in clinical applications. In our study, the observed decrease in surface roughness of soft liners containing 10% silver vanadate and 10% titanium dioxide may be attributed to the intrinsic properties of these nanoparticles and their interaction with the soft liner matrix. Both 10% silver vanadate and 10% titanium dioxide can act as fillers that occupy surface irregularities and enhance the homogeneity of the material. Additionally, their fine particle size allows for a smoother surface finish by minimizing porosity and reducing surface defects during the setting or curing process.

One of the more significant issues with soft denture liners is the failure of adhesion between the liner and the denture base. This bond failure can create a potential surface for bacterial growth, as well as plaque and calculus formation. Consequently, regular clinical evaluations and periodic replacement of soft denture liners are necessary to maintain oral health and denture function. These outcomes were attributed more to the high elasticity of the material than to issues with adhesion. The researchers suggested that the liner's failure was primarily due to its low cohesive strength, indicating the material’s internal weakness rather than accurately reflecting the bond strength between the liner and the denture base [8]. In the Dentsply Sirona Viscogel group, tensile bond strength was not statistically significant between the control group and experimental group with 10% titanium dioxide and between the experimental groups, thereby showing almost similar effects in terms of tensile bond strength. In the Maarc Dental Soft Liner group, the tensile bond strength of the soft liner incorporated with 10% silver vanadate was higher compared to both the control group and the experimental group with 10% titanium dioxide, which exhibited similar tensile bond strengths. The specific concentration of silver vanadate (10%) used in the study may have been optimal for enhancing bond strength without compromising the material's other properties. Exceeding this concentration could potentially lead to adverse effects, such as increased brittleness or reduced flexibility, which might negatively impact the tensile bond strength [16]. In the study by Avukat et al. [17], the results indicated that incorporating 1% by weight of titanium dioxide and hydroxyapatite nanoparticles into the soft liner material enhanced its bond strength. Titanium dioxide nanoparticles, in particular, exhibited strong surface interactions with the polymer matrix. Beyond enhancing the physical and optical characteristics of the polymer, they also offer resistance to environmental factors that contribute to material degradation, such as cracking and ageing [17].

Clinical implications

The incorporation of silver and titanium nanoparticles into soft denture lining materials offers a potential clinical advantage by reducing microbial colonization and infection risk beneath dentures. Since microorganisms can adhere to and penetrate the soft liner surface, leading to fungal and bacterial growth, nanoparticle-modified liners may help maintain oral tissue health and extend the service life of the prosthesis. The antimicrobial activity of silver, in particular, could minimize denture-related stomatitis and improve patient comfort without compromising the biocompatibility of the material.

Strengths

This study directly compared the antimicrobial efficacy of silver and titanium nanoparticles incorporated into soft denture liners under identical preparation and evaluation conditions, ensuring a reliable and valid comparative analysis. The methodology enabled simultaneous assessment of antifungal activity and mechanical properties, offering a comprehensive evaluation of the material’s clinical applicability. Standardized laboratory procedures and controlled testing conditions further enhanced the reproducibility and credibility of the results. Overall, this research adds valuable evidence to the limited literature on nanoparticle incorporation into soft denture liners and supports the development of modified materials with superior resistance to microbial colonization.

Limitations

The present study was conducted under in vitro conditions, which may not fully simulate the complex oral environment characterized by variations in saliva composition, pH, and temperature. Additionally, the antimicrobial activity was assessed without light activation, a factor that could influence the performance of TiO₂-based materials in clinical applications. Long-term parameters such as color stability and elasticity were not evaluated, which may affect the overall durability of the material in vivo. Therefore, further clinical trials are necessary to validate whether the antimicrobial benefits observed in this study translate effectively into improved oral health outcomes for denture wearers.

## Conclusions

Incorporation of 10% silver vanadate (AgVO₃) nanoparticles significantly enhanced the antifungal activity of Maarc Dental Soft Liner and Dentsply Sirona Viscogel against *Candida albicans*, producing a measurable inhibition zone, whereas the control and 10% titanium dioxide (TiO₂) groups showed no antifungal effect. The antifungal efficacy of AgVO₃ was statistically superior, particularly in the Viscogel group. Nanoparticle addition also affected mechanical properties: in Maarc Dental Soft Liner, TiO₂ increased hardness while AgVO₃ reduced it; in Viscogel, AgVO₃ increased hardness. Surface roughness decreased in all nanoparticle groups, with AgVO₃ producing the smoothest surface, especially in the Maarc Liner.

Tensile bond strength improved significantly with AgVO₃ in Maarc Dental Soft Liner but showed minor, non-significant reductions in Viscogel. TiO₂ had minimal influence on bond strength in both materials. Overall, 10% silver vanadate demonstrated superior antifungal activity and favorable modifications of physical properties compared to TiO₂, though the photocatalytic dependence of TiO₂ and the absence of light activation in this study indicate that results are condition-specific, warranting further molecular and clinical investigations.
